# Differentially expressed genes from RNA-Seq and functional enrichment results are affected by the choice of single-end versus paired-end reads and stranded versus non-stranded protocols

**DOI:** 10.1186/s12864-017-3797-0

**Published:** 2017-05-23

**Authors:** Susan M. Corley, Karen L. MacKenzie, Annemiek Beverdam, Louise F. Roddam, Marc R. Wilkins

**Affiliations:** 1 Systems Biology Initiative, School of Biotechnology and Biomolecular Sciences, UNSW Australia, Sydney, New South Wales Australia; 2 Children’s Cancer Institute Australia, Kensington New South Wales, Sydney, Australia; 3 School of Medical Sciences, UNSW Australia, Sydney, New South Wales Australia; 4 The School of Biomedical Sciences, The University of Queensland, Brisbane, Australia; 5 School of Medicine, University of Tasmania, Tasmania, Australia

**Keywords:** RNA-Seq, Transcriptomics, Paired-end reads, Single-end reads, Differential expression, Strand-specific, Non-strand-specific

## Abstract

**Background:**

RNA-Seq is now widely used as a research tool. Choices must be made whether to use paired-end (PE) or single-end (SE) sequencing, and whether to use strand-specific or non-specific (NS) library preparation kits. To date there has been no analysis of the effect of these choices on identifying differentially expressed genes (DEGs) between controls and treated samples and on downstream functional analysis.

**Results:**

We undertook four mammalian transcriptomics experiments to compare the effect of SE and PE protocols on read mapping, feature counting, identification of DEGs and functional analysis. For three of these experiments we also compared a non-stranded (NS) and a strand-specific approach to mapping the paired-end data. SE mapping resulted in a reduced number of reads mapped to features, in all four experiments, and lower read count per gene. Up to 4.3% of genes in the SE data and up to 12.3% of genes in the NS data had read counts which were significantly different compared to the PE data. Comparison of DEGs showed the presence of false positives (average 5%, using voom) and false negatives (average 5%, using voom) using the SE reads. These increased further, by one or two percentage points, with the NS data. Gene ontology functional enrichment (GO) of the DEGs arising from SE or NS approaches, revealed striking differences in the top 20 GO terms, with as little as 40% concordance with PE results. Caution is therefore advised in the interpretation of such results. By comparison, there was overall consistency in gene set enrichment analysis results.

**Conclusions:**

A strand-specific protocol should be used in library preparation to generate the most reliable and accurate profile of expression. Ideally PE reads are also recommended particularly for transcriptome assembly. Whilst SE reads produce a DEG list with around 5% of false positives and false negatives, this method can substantially reduce sequencing cost and this saving could be used to increase the number of biological replicates thereby increasing the power of the experiment. As SE reads, when used in association with gene set enrichment, can generate accurate biological results, this may be a desirable trade-off.

**Electronic supplementary material:**

The online version of this article (doi:10.1186/s12864-017-3797-0) contains supplementary material, which is available to authorized users.

## Background

Technical advances in next generation sequencing over the past decade have resulted in greater output of sequence data, and at a lower cost [1]. At the same time, analysis methods for understanding and interrogating sequence data have flourished [2–4]. This has resulted in the widespread uptake of techniques such as RNA-Seq for projects both large and small. RNA-Seq typically involves sequencing RNA obtained from a sample, quantification by mapping reads to genomic features, and comparison between conditions. Unlike micorarrays, no prior knowledge of samples (or probes) is necessary and hence it is possible to identify both known and novel transcripts as well as assembling a transcriptome de novo [5, 6]. The use of RNA-Seq to better understand the transcriptome of a vast range of organisms has grown dramatically over the past few years. With more researchers undertaking transcriptomic analyses, questions arise as to the most accurate and cost efficient way of doing this.

One question that arises is whether it is necessary to perform paired-end sequencing or whether single-end sequencing is adequate. Paired-end sequencing involves the sequencing of both ends of each cDNA fragment rather than sequencing only one end [7]. As the gap size between the ends of the fragment can be estimated, this technique facilitates accurate alignment back to the reference genome [8]. However, paired-end sequencing involves twice as much sequencing and is therefore more costly than single-end. So the decision to use any method will affect the number of samples which can be sequenced within a researcher’s budget. The number of biological replicates sequenced will affect the power of the experiment to find differential expression, which is the purpose of many RNA-Seq experiments [9].

Early RNA-Seq library preparation protocols could not determine the strand of origin and thus direction of any RNA read from the genome. This is problematic when there are overlapping genomic features. A number of techniques have now been developed to address this shortcoming, as reviewed by Levin et al. [10]. Strand-specific library preparation protocols, such as the Illumina TruSeq Stranded Total RNA Prep Kit or the Illumina TruSeq Stranded mRNA Library Prep Kit, are available and are no more costly than using non-strand aware protocols. There have been some recent analyses of strand-specific and non-strand-specific protocols, which point to the benefits of strand-specific approaches [11, 12].

We have used four different mammalian RNA-Seq experiments to assess the effect of using paired-end or single-end reads and a strand-specific versus non-specific library preparation protocol. We have looked at the effect of these factors on mapping, feature counting and on the ultimate objective of many RNA-Seq experiments – the calling of differentially expressed genes. We also consider the impact on functional insights which emerge from the differential expression analysis.

## Results

### Four mammalian RNA-Seq experiments using different read mapping strategies

Four different mammalian RNA-Seq experiments, detailed in Table 1, were used to study the effect of using single-end or paired-end reads in gene expression analysis. Paired-end data was also used to explore the difference that a strand-specific protocol can make compared to a non-strand-specific approach. Each of the four RNA-Seq experiments had a simple but typical design, comprised of three biological controls and three treated samples. The six samples in each of the experiments were independent biological replicates. Experiments 1 and 2 were from mouse tissue or primary cells whereas Experiments 3 and 4 involved human primary cells and a cell line respectively. In all four experiments sequencing was performed on both ends of the cDNA fragment (paired-end reads). We mapped both ends (see Methods) to produce our paired-end data set (PE data). We also mapped only the first read to produce our single-end read datasets for each experiment (SE data). Sequencing for Experiment 1 occurred in 2012 and used a non-strand-specific protocol for library preparation (Illumina TruSeq kit). The other three experiments were sequenced more recently with the Illumina TruSeq Standed library preparation kit [13]. For these three experiments we looked at the effect of analyzing the paired-end data with a protocol that recognizes the strand-specific nature of the reads (PE data) and also with a protocol that does not recognize this (NS data). In this way we could assess the difference that a strand-specific protocol makes to gene expression analysis.

**Table 1:** RNA-Seq data sets

Experiment	1	2	3	4
Species	*Mus musculus*	*Mus musculus*	*Homo sapiens*	*Homo sapiens*
Sample	Lip tissue	Keratinocytes (primary)	Hematopoietic cells (primary)	NuLi cells (cell line)
Control group (n)	3	3	3	3
Treated group (n)	3	3	3	3
Platform	Illumina HiSeq2000	Illumina NextSeq	Illumina NextSeq	Illumina NextSeq
Reads PE^a^ (average^d^)	35.3 M	37.4 M	84.7 M	37.2 M
Reads SE^b^ (average^d^)	35.0 M	38.2 M	85.3 M	37.5 M
Reads NS^c^ (average^d^)		37.5 M	84.9 M	37.3 M

^a^PE, paired-end reads

^b^SE, single-end reads

^c^NS, non-strand specific protocol for the paired-end reads

^d^Total number of reads from featureCounts summary (assigned plus non-assigned) with average of totals taken over the six samples in each experiment

### The number of assigned reads, multimapped reads and ambiguous reads varied between the PE, SE and NS data analyses

We mapped the reads to the respective genomes using Tophat2 [14] and then mapped the read loci to RNA features using the featureCounts function of Subread [15]. The featureCounts function gives the overall number of assigned reads as well as the number of reads which could not be assigned to RNA features because they were (i) ambiguous, (ii) multimapped or (iii) did not correspond to an RNA feature.

**Fig. 1:**
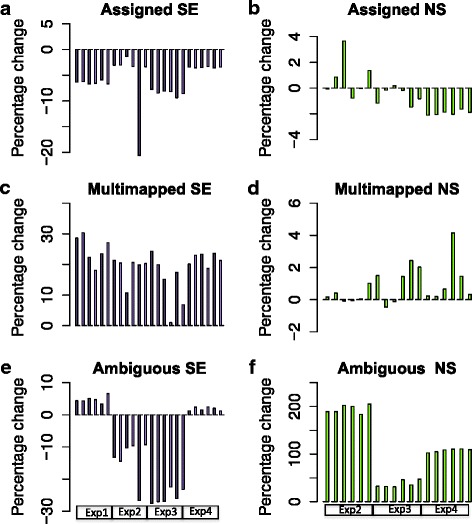
Percentage change in Assigned reads, Multimapped reads and Ambiguous reads. Barplots of the percentage change in reads assigned by featureCounts in the single-end (SE, *purple bars*) compared to the paired-end strand-specific data (PE) in a total of 24 samples from four experiments (Exp1, Exp2, Exp3, Exp4) and percentage change in non-strand-specific paired-end data (NS, *green bars*) compared to the paired-end strand-specific data (PE) in a total of 18 samples from three experiments (Exp2, Exp3, Exp4). **a** Assigned reads, SE comparison, (**b**) Assigned reads, NS comparison (**c**) Multimapped reads, SE comparison (**d**) Multimapped reads, NS comparison (**e**) Ambiguous reads, SE comparison (**f**) Ambiguous reads, NS comparison

In all experiments, the use of SE as opposed to PE reads resulted in a reduction in the number of reads that could be uniquely assigned to an RNA feature (Fig. 1a). The reduction ranged from 3.3% to 9.4% with one outlier, sample 5 in experiment 2 differing by 20%. Comparison of the PE reads with the paired-end non-strand-specific data (NS reads) was possible for Experiments 2, 3 and 4. Interestingly, this analysis did not show a consistent difference across the three experiments (Fig. 1b). Some samples in Experiments 2 and 3 showed a small reduction in the number of assigned reads in the NS data (less than 3%) whereas other samples showed a small increase in the number of assigned reads (less than 4%). By contrast, Experiment 4 showed a small but consistent decrease in the number of assigned reads in the NS data (about or less than 2%).

We investigated the reduction in the number of reads uniquely assigned using SE mapping. This revealed a high proportion of reads being ‘multimapped’, with an average increase of 20% of multimapped reads compared to the PE data (Fig. 1c). This indicates that many 75 bp reads are not sufficiently unique to allow mapping to a unique genomic loci whereas the availability of the other end of the DNA fragment enables the exact genomic location of the fragment to be identified. When comparing the strand-specific PE and NS data, there was little difference in the number of multimapped reads (Fig. 1d). However, in this case we saw a striking increase in the number of ambiguous reads ranging from a 200% increase in Experiment 2 to a 40% increase in Experiment 3, with an average increase of 116% over the three experiments (Fig. 1f). By contrast we saw a decrease in ambiguous reads in the SE data in Experiments 2 and 3 and a small increase < 10% in Experiments 1 and 4 (Fig. 1e). Ambiguous reads arise when the genomic location of a read is known but where that location may be part of more than one gene or other feature, on the same or opposite strand of DNA. Using a strand-specific protocol resolves ambiguity arising from the latter, and hence we see the least ambiguous reads using the strand-specific protocol.

### Read count per gene tends to be less in SE data compared to PE data

**Fig. 2:**
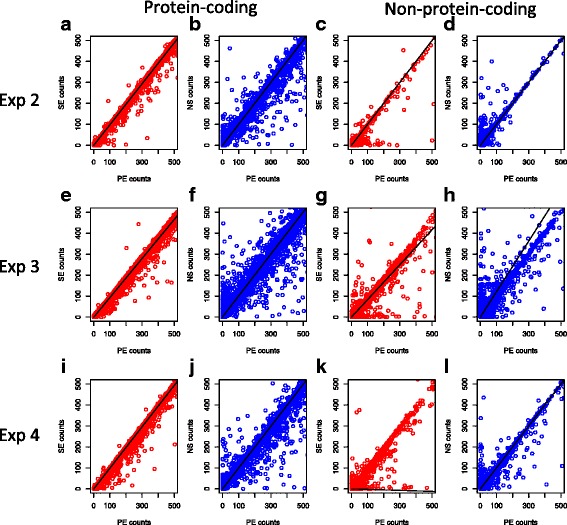
Scatterplots of reads assigned to protein coding features and non-coding features. Counts for all protein coding genes features (**a**, **b**, **e**, **f**, **i**, **j**) and for all non-coding features (**c**, **d**, **g**, **h**, **k**, **l**) are compared for the PE data versus the SE data (*red*) and the PE data using the strand specific protocol with the paired end data using a non-strand specific protocol (NS data, *blue*). Counts for the paired-end strand specific datasets (PE) are on the x-axis in all cases. Data is shown for sample 1 in Experiment 2 (row 1), sample 1 in Experiment 3 (row 2) and sample 1 in Experiment 4 (row 3)

We examined the number of reads per gene, in the SE data compared to the PE data, to determine if there were any differences in counts. Scatterplots of one sample from each experiment illustrate a systematic tendency for the counts per gene to be less in the SE data compared to the PE data (Fig. 2, red plots). By contrast, the comparison of the PE and NS data (Fig. 2, blue plots) showed more variation in the counts. There was also evidence that the non-strand-specific approach allocates counts to features that have no counts in the strand-specific approach, as seen by the large number of points mapping close to the y-axes of these plots. That is, in some cases the non-strand-specific approach will indicate that genes or non-coding features are expressed when they are not.

### The number of assigned reads are significantly different in SE and NS data compared to PE data

We next investigated whether the different sequencing methods resulted in changes in the number of assigned reads. To test for statistically significant differences, in the counts from SE or NS data compared to the PE data, we used the statistical tools available in edgeR. A differential expression test was used to compare the read counts per gene in the controls generated with the PE data versus the reads for the controls in the SE and NS data sets. Ideally, we should find no difference between the groups if each sequencing method produces similarly reliable read count data. Any difference identified indicates the degree to which alternate sequencing methods produce unreliable count data. We performed this comparison using edgeR, as described in Methods. Applying a multitest correction (FDR < 0.05) we found that between 111 and 608 genes (equating to 0.72 – 4.29% of genes tested) had significantly different counts when comparing the PE and SE data (Table 2). A high proportion of the genes were apparently down-regulated (Exp 1, 80%; Exp 2, 94%; Exp 3, 88%; Exp 4, 93%). This is consistent with the trend we saw of a lower number of read counts per gene when using the SE protocol (Fig. 2). Comparing the NS and PE data we find that the number of differentially expressed genes was between 729 and 1719 (equating to 5.74 to 12.26% of genes tested) (Table 2). The distribution between down-regulated and up-regulated genes was more even in this case, with down-regulated genes being 50–75% (Exp 2, 57%; Exp 3, 50%; Exp 4, 75%).

**Table 2:** Number of genes with significantly different counts

Experiment	1	2	3	4
Genes tested	15456	12702	14016	14166
*biotype*				
protein coding	14328 (92.7%)	11891 (93.6%)	12009 (85.7%)	12530 (88.5%)
non protein coding	1027 (6.6%)	733 (5.8%)	1988 (14.2%)	1620 (11.4%)
- pseudogenes	232	145	452	396
- antisense	242	124	604	532
SEvsPE DEGs^a^	111 (0.7%)	176 (1.4%)	327 (2.3%)	608 (4.3%)
*biotype*				
protein coding^b^	29 (26.1%)	93 (52.8%)	139 (42.5%)	396 (65.1%)
non protein coding^c^	82 (73.9%)	74 (42%)	186 (56.9%)	208 (34.2%)
- pseudogenes^d^	76 (68.5)	50 (28.4%)	105 (32.1%)	150 (24.7%)
- antisense^e^	1 (0.9%)	3 (1.7%)	13 (4.0%)	12 (2.0%)
NSvsPE DEGs^f^	NA	729 (5.74%)	1719 (12.26%)	1615 (11.4%)
*biotype*				
protein coding^b^		555 (76.1%)	1167 (67.9%)	1291 (79.9%)
non protein coding^c^		167 (22.9%)	543 (31.6%)	317 (19.6%)
- pseudogenes^d^		15 (2.1%)	79 (4.6%)	45 (2.8%)
- antisense^e^		48 (6.6%)	271 (15.8%)	167 (10.3%)

^a^ Differential expression (DE) test of Controls in PE data (*n* = 3) versus Controls in SE data (*n* = 3) using edgeR, FDR < 0.05

^b^ Number of DE protein coding genes and proportion as a percentage of the tested genes

^c^ Number of DE non-protein-coding genes and proportion as a percentage of the tested genes

^d^Number of DE pseudogenes

^e^ Number of DE genes

^f^ DE test of Controls in PE data (*n* = 3) versus Controls in NS data (*n* = 3) using edgeR, FDR < 0.05

**Fig. 3:**
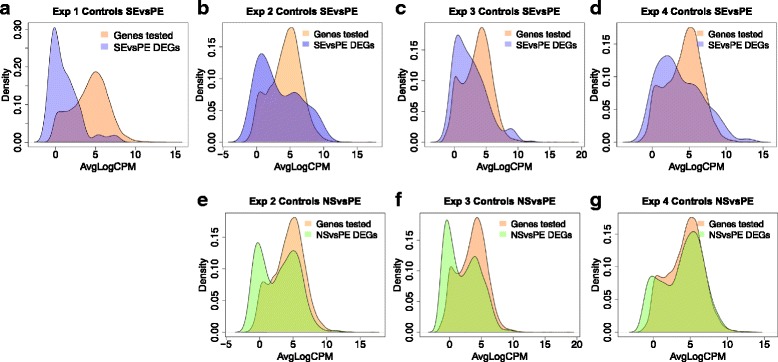
Density plots comparing distribution of counts in the differentially expressed genes with the distribution of counts for all genes expressed in the controls. The distribution of average read counts (AveLogCPM) for each of the tested genes (as calculated in edgeR) is plotted (*orange*). **a**-**d** The distribution of AveLogCPM counts for the differentially expressed genes found in the SE vs PE comparison are superimposed (*purple*) for Experiments 1–4. The overlapping region is a combination of *orange* and *purple*. **e**-**g** The distribution of AveLogCPM counts for the differentially expressed genes found in the NS vs PE comparison are superimposed (*green*) for Experiments 2–4. The overlapping region is a combination of *orange* and *green*

We further explored the characteristics of the gene counts that differed between data sets. For this, we created density plots of the average counts (average logCPM) for all genes expressed in the controls and compared these to the counts for genes that were significantly different between SE and PE data, or between the NS and PE data (Fig. 3). In both comparisons we saw that significantly differentially expressed genes could be of high, intermediate or low expression levels. Although the range of gene expression levels was covered, we saw a peak in the low expression range indicating that differentially expressed genes were more lowly expressed in the SE versus PE data (Fig. 3a-d). In the NS versus PE data, the differentially expressed genes showed a bimodal distribution with a peak also in the high expression range (Fig. 3e-g).

### Proportion of protein-coding and non-coding reads is affected by seqeuncing protocols

**Fig. 4:**
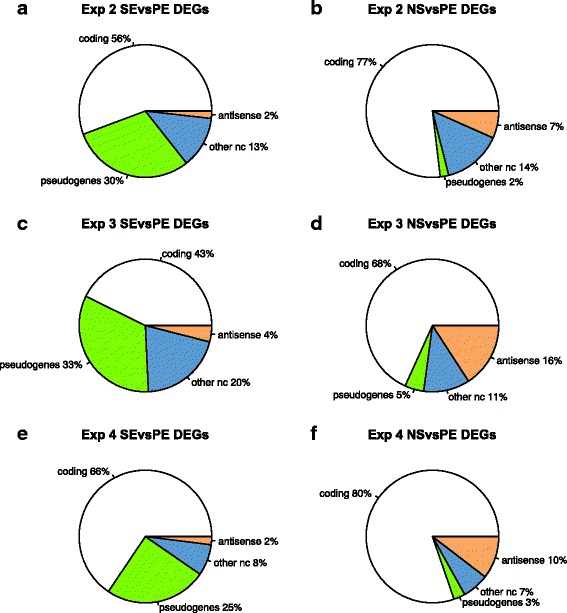
Pie charts of differentially expressed genes (DEGs) showing proportion of protein coding and non-protein coding features. The DEGs are classified as: protein coding (*white*), pseudogenes (*green*), antisense (*orange*), non-coding RNAs, other than pseudogenes and antisense (*blue*). DEGs found in the SE vs PE comparison are in column 1 (**a**, **c**, **e**) and DEGs found in the NS vs PE comparison are in column 2 (**b**, **d**, **f**)

It is known that non-coding RNA features tend to have lower expression than protein-coding genes. We therefore examined whether the allocation of reads between protein-coding and non-coding features were different in the SE data compared to the NS data. We saw that the non-coding transcripts were over-represented in the DEG lists in both the SE versus PE comparison and the NS versus PE comparison. As shown in Fig. 4 the proportion of non-coding DEGs ranged between 20% (Exp 4 NSvsPE, Fig. 4f) and 57% (Exp 3 SEvsPE, Fig. 4c). This was much higher than the genes tested for differential expression which, after filtering of lowly expressed genes, comprised between 6 and 11% of non-coding transcripts (Table 2). Accordingly, we see that the quantification of non-coding transcripts is disproportionately affected by sequencing method. This effect was more pronounced in the SE data compared to the NS data where we saw that a high proportion of the non-coding genes are pseudogenes, (between 25% and 69% of the DEGs generated in the SE vs PE comparison are pseudogenes). By contrast, the proportion of pseudogenes found to be significantly different in the NS vs PE comparison ranged from 2–5% of the DEGs. Reads mapping to pseudogenes are also likely to map to an ancestral gene. These reads are therefore prone to be ‘multimapped’ and discarded using single-end sequencing, however, it appears that this effect is lessened where both ends of the DNA fragment are available for mapping.

By contrast, the DEGs found with a non-strand-specific protocol have a higher proportion of antisense reads (ranging from 7–16% of the DEGs in the three experiments) compared to 1–4% of the DEGs in the SE vs PE comparison. This is also consistent with expectation as, by definition, antisense transcripts overlap genetic features on the opposite DNA strand and so are likely to be considered ‘ambiguous’ if a non-strand-specific protocol is used.

### Differential expression analysis of treated versus control samples

**Fig. 5:**
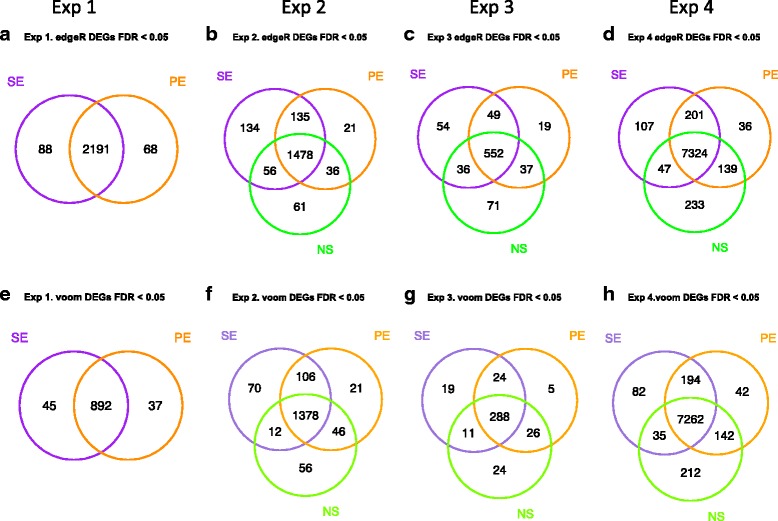
Differentially expressed genes (DEGs) identified in each of the four experiments. Venn diagrams of the DEGs comparing those derived from the PE data (*orange*) with those derived from the SE data (*purple*) and the NS data (*green*). Differentially expressed genes were identified using edgeR (**a**-**d**) and voom (limma) (**e**-**h**) with an FDR cut-off of 0.05. **a**, **e** Experiment 1, (**b**, **f**) Experiment 2, (**c**, **g**) Experiment 3, (**d**, **h**) Experiment 4

A critical question is whether the differences in read mapping are of consequence for the comparison of treated and control samples. To investigate this, we performed differential expression analysis of gene expression in the 3 treated samples versus the three controls in each of the four experiments. We performed this analysis using edgeR and the voom function of limma [16, 17], employing an FDR cut-off of 0.05 to identify differentially expressed genes (DEGs). The DEGs found using the PE, SE and NS data are compared in the Venn diagrams in Fig. 5(a-h). Assuming that the PE data is more accurate, these plots show that false positives and false negatives are seen using the alternative data. We found that the SE data generated false positives (average across four experiments (edgeR 7%, voom 5%)) and false negatives (average across four experiments (edgeR, 4%,voom, 5%)). The discrepancy was higher when comparing the PE data and NS data. False negatives were higher in all three experiments (average across three experiments (edgeR, 8%, voom 7%)), and false positives were higher in Experiment 3 and Experiment 4 (average false positives across three experiments (edgeR, 9%, voom, 6%)).

**Fig. 6:**
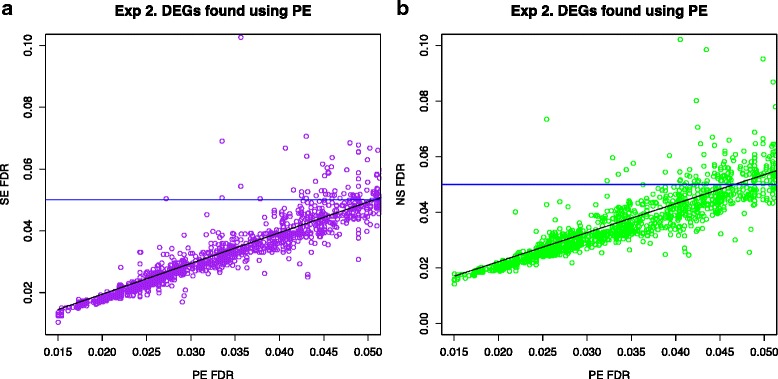
Comparing FDR values of the DEGs found using the PE data with the SE and NS data. The FDR values of the DEGs found in the PE data set are plotted on the x-axis and the corresponding FDR value for those genes in the SE data or NS data are plotted on the y-axis. The *blue horizontal line* shows the 0.05 cut-off. All genes above the line would not be classed as DEGs although the FDR is lower than 0.1. **a** Experiment 2 PE vs SE comparison (**b**) Experiment 2 PE vs NS comparison

The lists of DEGs, analysed above, were obtained by imposing a hard cut-off (here we use FDR < 0.05). However, there might be genes that are close to this cut-off that may be called as DE (or not) if the arbitrary cut-off is changed. To investigate this we plotted the FDR values for the DEGs found using the PE data in Experiment 2 against the FDR values for these genes found using the SE or NS data (Fig. 6). The genes not identified as being differentially expressed by the SE or NS method are those above the blue horizontal line and have an FDR in the range 0.05 to 0.1. These would have been included if a cut-off of 0.1 had been chosen, but the same effect would have been apparent with another set of genes close to the new cut-off.

### Functional analysis of differentially expressed genes using gene ontology (GO) analysis and gene set enrichment

Given that different sequencing methods were affecting the genes found to be differentially expressed in the treated samples versus controls, we investigated whether this then affected functional analysis results. We first used the goana function [18] in the limma Bioconductor package to find the most enriched gene ontology terms in the lists of DEGs. For each experiment we looked for the degree of concordance in the top ranked GO terms produced using SE, PE and NS lists of DEGs. These results are presented in Table 3. If only the top 20 GO terms were considered, there was a striking discrepancy in the functional enrichment results between sequencing protocols. For example, in Experiment 2 we found only 65% concordance for the SE vs PE comparison and just 40% concordance for the NS vs PE comparison. If we took a larger group of GO terms the concordance improved. Taking the top 300 GO terms associated with the DEGs, the concordance improved to 85–99% concordance for the PE versus SE comparison and 80–96% concordance for the PE versus NS comparison. However the GO terms were not necessarily ranked in the same order and, as such, the degree of concordance varied if different sized sets of GO terms were selected solely on the basis of statistical ranking.

**Table 3:** Concordance for GO terms found using goana function

PE vs SE	Exp 1^a^	Exp 2^a^	Exp 3^a^	Exp 4^a^
Top 20 GO terms	95	65	85	100
Top 50	84	94	88	96
Top 100	79	91	94	98
Top 200	84	86	94	97
Top 300	88	87	85	99
PE vs NS		Exp 2^b^	Exp 3^b^	Exp 4^b^
Top 20 GO terms		40	75	95
Top 50		86	84	90
Top 100		88	91	96
Top 200		78	95	96
Top 300		80	88	96

^a^Percentage concordance in the top GO terms (by FDR) found using the goana function of limma when using the SE and PE datasets for each of the experiments

^b^Percentage concordance in the top 20 GO terms (by FDR) found using the goana function of limma when using the SE and PE datasets for each of the experiments

Gene ontology enrichment analyses are frequently based on lists of DEGs defined by use of a hard cut-off, such as an adjusted *P* value of 0.05. This is a common approach in RNA-Seq experiments. However, another way of approaching functional evaluation is to use gene set enrichment analyses, that consider all the genes tested in the differential expression analysis. We used the camera function [19] included in the limma package to perform this; it implements a competitive test to determine whether the genes in the gene set are more often differentially expressed than the remainder of genes not in the gene set. In this way it is possible to ascertain the order of importance of the various gene sets in the experimental condition of interest. We tested for concordance in the top gene sets identified by camera over the PE, SE and NS data (Table 4). Using the camera function we found a high concordance of 97–99% in the top 200 gene sets when comparing the PE and SE results over the four experiments. Similar results were obtained when comparing the PE and NS results with concordance ranging between 96% and 99% over the three experiments in the top 200 gene sets.

**Table 4:** Concordance for gene sets found in gene set analysis using the camera function

PE vs SE	Exp 1^a^	Exp 2^a^	Exp 3^a^	Exp 4^a^
Top 50 gene sets	100	98	96	98
Top 100	98	98	98	97
Top 200	99	99	97	99
Top 400	99	PE total 243	98	99
PE vs NS		Exp 2^b^	Exp 3^b^	Exp 4^b^
Top 50 gene sets		98	96	98
Top 100		97	94	93
Top 200		99	96	99
Top 400		PE total 243	96	96

^a^Percentage concordance in the top gene sets (by FDR) found using the camera function of limma when using the SE and PE datasets for each of the experiments

^b^Percentage concordance in the top top gene sets (by FDR) found using the camera function of limma when using the PE strand specific and non-strand specific datasets for experiments 2–4

### The importance of biological replicates

This study was based on four experiments, each of which used a 3 by 3 design (3 controls versus 3 treated samples). Such designs are typical in RNA-Seq experiments where researchers are constrained by cost and /or sample availability. It is known that additional replicates increase the power to identify differentially expressed genes 9 [20]. To understand the effect of adding more replicates in our RNA-Seq experiments, we carried out power analysis. We found that the power to detect genes with a two-fold expression change ranged from 61% (Experiment 1) to 76% (Experiment 4) with the 3 by 3 design. Interestingly, this could be increased by the addition of two more replicates per condition, to 82% in Experiment 1 and 93% in Experiment 4, that is, an increase in power of around 15% could be achieved with a 5 by 5 design (Table 5). The 3 by 3 design was underpowered to detect genes with a lower fold change, say of 1.5 and ranged from 26–35% over the four experiments. The power to detect these genes was also increased by a similar increment of around 15% by the addition of two replicates to each condition. The algorithm used to estimate power is based on initial estimates of biological variation in the samples and depth of sequencing. These parameters were similar in the PE and SE datasets (Table 5) and consequently the power calculations for the PE and SE data yield the same result for up to the third decimal place (Table 5).

**Table 5:** Power calculations using functions in RNASeqPower

	Depth^a^	PE CV^b^	SE CV^c^	FC^d^	PE Power (*n* = 3)^e^	SE Power (*n* = 3)^f^	PE Power (*n* = 5)^e^	SE Power (*n* = 5)^f^
Exp 1	10	0.2074	0.2069	1.5	0.2589	0.2592	0.3956	0.3961
				2	0.6121	0.6127	0.8259	0.8264
Exp 2	10	0.1052	0.1007	1.5	0.3192	0.3215	0.4855	0.4887
				2	0.7215	0.7251	0.9080	0.9103
Exp 3	10	0.099	0.0907	1.5	0.3223	0.3262	0.4899	0.4955
				2	0.7264	0.7325	0.9111	0.9149
Exp 4	10	0.0264	0.0256	1.5	0.3464	0.3465	0.5241	0.5242
				2	0.7628	0.7630	0.9324	0.9325

^a^Average depth of coverage

^b^Biological coefficient of variation derived from the estimateCommonDispersion function of edgeR for the PE data

^c^Biological coefficient of variation derived from the estimateCommonDispersion function of edgeR for the SE data

^d^Fold change

^e^The power calculations performed for the PE data using the RNASeqPower package

^f^The power calculations performed for the SE data using the RNASeqPower package

SE data can be produced more economically than PE and can potentially allow the use of additional replicates. To investigate this, we tested whether differential expression analysis between controls and treated samples differed when using 3 biological replicates per condition with SE data compared to using 2 biological replicates per condition with PE data. In all cases we found that using 3 biological replicates with SE data was superior to using 2 biological replicates with PE data for identifying DEGs, as judged against the gold standard for this experiment (three biological replicates with PE). However, it should be noted that our results were influenced by which 2 biological replicates were chosen for study, indicating the inadequacy of 2 replicates for estimating biological variation. These data are presented in Additional file 1, Additional file 2: Table S1 and Additional file 3: Figure S1. The use of only 2 biological replicates is extreme, and is not recommended, however this analysis does indicate the benefit of increasing the number of biological replicates even though this may be at the expense of sequencing both ends of the RNA-seq reads.

## Discussion

RNA-Seq technology has been keenly embraced by the research community, as evidenced by the hundreds of publications involving the deep sequencing and analysis of transcriptomes. Sequencing centres generally offer a number of library preparation strategies, along with paired-end or single-end sequencing. Initially, sequencing chemistry could not distinguish the DNA strand from which a read originated. However, straightforward strand-specific chemistry has become available recently, such as that provided by the Illumina TruSeq Stranded Library Prep Kits, as used in this study. Sequencing centres and researchers about to embark upon an RNA-Seq project require information to make decisions regarding library preparation. This motivated us to undertake this comparison, involving four mammalian RNA-Seq experiments.

Single-end sequencing involves half the amount of sequencing as paired-end sequencing and thus halves the sequencing cost, excluding sample preparation. Based on this it is an attractive option. However, this must be balanced against its drawbacks. Our study shows that single-end reads, compared to paired-end sequencing, result in a reduction in the number of reads that can be assigned to RNA features and a trend of lower read counts per feature. As a consequence, between 0.72% and 4.29% of expressed genes had significantly different counts when comparing the SE data to PE data. The main reason for this appears to be the discarding of multimapped reads which reduce the reads that could be assigned to features. We found that this had a strong effect on read counts for pseudogenes but also affected other non-protein coding features as well as protein coding genes.

Using a non-strand-specific protocol had an even greater impact on results. Up to 12.26% of the tested genes showed significantly different counts in the NS data, as compared to the PE data. Non-strand specific protocols therefore pose a definite risk to accurate analysis. An earlier study comparing a stranded and non-stranded RNA-Seq protocol in blood samples from five human subjects found 10.65% of genes were differentially expressed when comparing these protocols [12]. Our study gave similar results in the human samples (12.26%, Exp 3 and 11.40%, Exp 4). Whilst SE data showed a trend towards decreased read counts per gene, the NS data was more variable with read counts being higher for some genes but lower for others. It is interesting though that many genes had reads allocated in the NS protocol but no counts using the strand-specific data. This spurious allocation of reads is likely to lead to the incorrect assumption that certain features are expressed when they are not. Indeed it has previously been observed that a non-strand-specific protocol results in a significant fraction of genes having overestimated expression values; this poses a significant problem given that approximately 16% of protein coding genes are overlapping [13].

Differential expression analysis of the controls and treated samples, using PE data as a gold standard, revealed that the NS data produces a greater proportion of false positives and false negatives than occurs using the SE data. We found that this had a non-negligible effect on evaluating differential expression. In RNA-Seq analyses it is usual to derive a list of DEGs based on a threshold statistical value, such as a multitest adjusted *p*-value of 0.05. Adopting this standard approach single-end data produced both false positives (average across four experiments (edgeR, 7%, voom, 5%), and false negatives (average across four experiments (edgeR, 4%, voom, 5%)). Differential expression analysis of the controls and treated samples, using PE data as a gold standard, revealed that the NS data produces a greater proportion of false positives and false negatives than occurs using the SE data (average false positives across three experiments (edgeR, 9%, voom, 6%) and average false negatives across three experiments (edgeR, 8%, voom, 7%).

The utility of single-end reads may depend on the questions being asked in the research. When it comes to understanding any functional differences between case and controls in the experiment, we saw that SE reads and NS reads can lead to a dramatic difference in the top 20 gene ontology terms arising from enrichment analysis. For example in Experiment 2, there was only 65% concordance in GO terms found between the SE and PE data and 40% concordance between the NS and PE data. Caution is thus to be advised if using SE data or NS data. However, there was reasonable agreement between the top 300 GO terms identified from the DEGs. If a comprehensive list of GO terms is used it is therefore likely that the same broad conclusions would be drawn as to the functional effect of the treated samples versus the controls. Interestingly, when we supplemented the use of DEGs by a gene set enrichment analysis approach, which uses all tested genes, we found strong agreement in the most significant gene sets. In this case, the same biological conclusions could be drawn regardless of the sequencing and read mapping method.

A final consideration when undertaking RNA-Seq is the power to detect differentially expressed genes. This increases with additional replicates. We estimate that power to detect differential expression could be increased by around 15% if the number of biological replicates in each condition was increased from three to five. As cost of sequencing is an important factor in experimental design it may be that the combination of single-end reads with increased number of biological replicates would be a sound trade-off, especially if care is taken in the functional analysis of results. Our analysis indicates that using 3 biological replicates per condition with SE is preferable to using 2 biological replicates with PE sequencing when undertaking differential expression analysis. This is a rather extreme comparison, however it does illustrate the important advantages that can be gained with additional biological replicates.

There is no cost difference in using a strand-specific and non-strand-specific library preparation. In this case it is clear that a strand-specific method is preferable. A strand-specific protocol avoids underestimation of read counts as occurs when a read could be allocated to alternative features. The number of ambiguous reads decreases and a strand-specific protocol avoids spurious allocation of reads to features in cases where the feature is not actually expressed.

## Conclusions and recommendations

Using a paired-end strand-specific protocol is necessary to obtain an accurate read count for all genomic features. Errors in read counts will occur from use of single-end or non-stranded sequencing, and lead to false negatives and false positives in the analysis of differentially expressed genes. This can and will affect downstream analysis, including in functional GO enrichment analysis. Ultimately, this can affect the biological interpretation of results.

At the same time it must be borne in mind that using SE mapping reduces the sequencing cost and that this saving could be used to increase the number of biological replicates. This will increase the power of an experiment, and may be a desirable trade-off. There is no similar advantage in using a non-strand-specific protocol and we would always recommend the use of a strand-specific protocol.

In conclusion, the use of a strand-specific protocol is recommended in all cases. The use of single-end reads with additional replicates may be preferable to paired-end reads with less replicates for differential expression analysis. However, if transcriptome assembly is the primary objective of an experiment then use of paired-end reads will be a better strategy.

## Methods

### Samples

Experiments 1 and 2 involved the use of mice. All experimental procedures were approved by the Animal Care and Ethics Committee at UNSW Australia.

Experiment 3 involved the use of human primary hematopoietic cells. All experimental procedures were approved by the Human Research Ethics Committee and Institutional Biosafety Committee at UNSW Australia.

Experiment 4 involved the use of a human NuLi cell line [21] and did not require ethics approval.

### Transcriptome sequencing

mRNA libraries for all four experiments were prepared at the Ramaciotti Centre for Genomics (UNSW Australia). The Illumina TruSeq RNA Prep Kit was used for Experiment 1. The Illumina TruSeq Stranded Total RNA Prep Kit was used for Experiments 2-3 and the Illumina TruSeq Stranded mRNA Prep Kit was used for Experiment 4. The six RNA-Seq libraries in Experiment 1 were sequenced on the Illumina HiSeq2000 platform, Experiments 2, 3 and 4 were sequenced using the Illumina NextSeq 500. R1.fastq and R2.fastq files were produced for each sample.

### Mapping RNA-Seq reads

The reads for Experiment 1 and Experiment 2 were mapped to the Ensembl *Mus musculus* genome (GRCm38). The reads for Experiment 3 and Experiment 4 were mapped to the Ensembl *Homo sapiens* genome (GRCh38). Mapping was performed with Tophat2 (v 2.0.12) [14] calling Bowtie2 (v 2.1.0) [22]. For paired-end mapping we used the settings: tophat2 -p 6 --library-type fr-firststrand -G $gtf -o $output $ref *R1*.fastq.gz *R2*.fastq.gz. For single-end mapping we used the settings: tophat2 -p 6 --library-type fr-firststrand -G $gtf -o $output $ref *R1*.fastq.gz. To process in a non-strand-specific manner we used the settings: tophat2 -p 6 --library-type fr-unstranded -G $gtf -o $output $ref *R1*.fastq.gz *R2*.fastq.gz.

The featureCounts function of Subread [15] was used to generate counts of reads uniquely mapped to annotated genes using the GRCm38 annotation gtf file and the GRCh38 annotation gtf file respectively. For the bam files produced from the strand-specific paired-end data we used the script: featureCounts -s 2 -T 12 -p -a $gtf -t exon -g gene_id -o $seq\featurecounts.txt n_sort.accepted_hits.bam. For the bam files produced from the strand-specific single-end data we used the script: featureCounts -s 2 -T 12 -a $gtf -t exon -g gene_id -o $seq\featurecounts.txt n_sort.accepted_hits.bam. For the non-strand-specific protocol we used the script featureCounts -s 0 -T 12 -p -a $gtf -t exon -g gene_id -o $seq\ featurecounts.txt n_sort.accepted_hits.bam.

### Differential gene expression analysis

Tables of raw counts generated using featureCounts were used as input in all analyses. Comparison of controls in the PE, SE and NS datasets was performed using edgeR (v3.14.0). We excluded lowly expressed genes and tested those genes with expression of at least 1 CPM (counts per million) in at least one of the controls. The number of genes retained for testing in each of the experiments was as follows: Experiment 1: 15456, Experiment 2: 12702, Experiment 3: 14016, Experiment 4: 14166. Counts were normalized using the TMM method and generalized linear models were used for differential expression analysis. Comparison of the controls versus treated samples in each of the four experiments was performed using functions in the edgeR (v 3.14.0) [23] and limma (v 3.28.17) [17] Bioconductor packages. Low count transcripts were excluded and only those genes with at least 1 count per million (cpm) in at least 3 samples were used for analysis. In all cases differentially expressed genes were defined as those genes with a Benjamini-Hochberg corrected *p* value less than 0.05.

### Functional analysis

We used the goana function [18] included in the limma Bioconductor package to find the most enriched gene ontology terms in the lists of DEGs. The Gene Ontology (GO) terms in the categories Biological Process, Cellular Component and Molecular Function were included. The false discovery rate (FDR) was set to 0.05 and the function topGO was used to order the GO terms by statistical significance. We compared sets comprised of the top 20, 50, 100, 200 and 300 GO terms.

We used the camera function [19] included in the limma package to perform gene set enrichment analysis. We interrogated the gene sets contained in mouse_c2_v5.rdata for mouse and human_c2_v5.rdata for human downloaded from http://bioinf.wehi.edu.au/software/MSigDB. We used an inter.gene.cor = 0.01 and an FDR value of 0.01. We compared sets comprised of the top 20, 50, 100, 200 and 300 GO terms.

### Power calculations

We carried out power calculations using the RNASeqPower package [24]. The required inputs to the rnapower function are the coefficient of variation between biological replicates (CV) and average read depth per gene. We calculated the biological co-efficient of variation in each experiment using the function estimateGLMCommonDisp from edgeR. We used an average depth of 10 reads and confirmed this estimate by creating multidensity plots of count distribution.

## Additional files

Additional file 1: Comparing differential expression analysis using 2 biological replicates of the PE data versus using 3 biological replicates of the SE data. This document contains the DE analysis of control versus treated samples conducted using only 2 biological replicates from the PE data set compared to results obtained using 3 biological replicates from the SE data set. (DOCX 184 kb)

Additional file 2: Table S1. Numbers of DEGs found using 2 biological replicates with PE and 3 biological replicates with SE. (XLSX 42 kb)

Additional file 3: Figure S1. Venn diagrams of the DEGs comparing 3 biological replicates from the SE data with 2 biological replicates from the PE data. (PPTX 237 kb)
