# Supplementary material for: Differentially expressed genes from RNA-Seq and functional enrichment results are affected by the choice of single-end versus paired-end reads and stranded versus non-stranded protocols
Source: BMC Genomics. 2017 May 23;18:399. doi: 10.1186/s12864-017-3797-0 (PMC5442695; doi:10.1186/s12864-017-3797-0)
Supplement: Supplementary file 1 — Comparing differential expression analysis using 2 biological replicates of the PE data versus using 3 biological replicates of the SE data. This document contains the DE analysis of control versus treated samples conducted using only 2 biological replicates from the PE data set compared to results obtained using 3 biological replicates from the SE data set. (DOCX 184 kb) [file 12864_2017_3797_MOESM1_ESM.docx]

**Additional File 1**

**Investigation of tradeoff between increased replicates SE sequencing and lower replicates with PE sequencing**

In circumstances where funds or samples are limited, it is important to understand whether the analysis of additional replicates or the use of PE over SE sequencing is preferable. To understand this, we compared the differential expression analysis of controls versus treated samples in all four experiments using only two biological replicates from the PE data versus three biological replicates from the SE data. Differentially expressed genes were identified using voom (limma) with an FDR cut-off of 0.05 using the same protocol for DE analysis described in Methods.

We performed this experiment using replicates 1 and 2 in each of the four experiments and then using replicates 2 and 3 in each of the four experiments.

The results presented in Table S1 show that there is considerable difference depending on which two of the three replicates are chosen. Use of replicates 1 and 2 is labeled as “PE_2”. Use of replicates 2 and 3 is labeled as “PE_2.2”.

We see that when only two biological replicates are used we may find no or few differentially expressed genes (see Table S1: Exp 2. PE_2 and Exp 3. PE_2.2). Where we find a reasonably high number of the DEGs (say over 85%) we also find a high number of false positives (see Table S1: Exp 1. PE_2 and Exp 4, PE_2).

In all cases using three biological replicates with SE was superior to using only two biological replicates with PE sequencing as judged against the gold standard for this experiment (three biological replicates with PE).

This indicates the importance of having agreater number of biological replicates. Other studies have reported the positive effect of increasing the number of replicates on the ability to call differential expression.

**Table S1: Comparing numbers of DEGs found using 2 biological replicates with PE and 3 biological replicates with SE**

| **Exp 1** | **DEGs** | **True positives** | **True positives (% )** | **False positives** | **False positives (% SE)** |
| --- | --- | --- | --- | --- | --- |
| PE (3 reps) | 929 | 929 |  | 0 |  |
| SE (3 reps) | 937 | 892 | 96 | 45 | 5 |
| PE_2 (reps 1,2) | 3305 | 912 | 98 | 2393 | 72 |
| PE_2.2 (reps 2,3) | 325 | 306 | 33 | 19 | 6 |
| **Exp 2** | **DEGs** | **True positives** | **True positives (%)** | **False positives** | **False positives (% SE)** |
| PE (3 reps) | 1551 | 1551 |  | 0 |  |
| SE (3 reps) | 1566 | 1484 | 96 | 82 | 5 |
| PE_2 (reps 1,2) | 0 | 0 | 0 | 0 |  |
| PE_2.2 (reps 2,3) | 613 | 501 | 32 | 112 | 18 |
| **Exp 3** | **DEGs** | **True positives** | **True positives (% )** | **False positives** | **False positives (% SE)** |
| PE (3 reps) | 342 | 342 |  | 0 |  |
| SE (3 reps) | 341 | 311 | 91 | 30 | 9 |
| PE_2 (reps 1,2) | 92 | 87 | 25 | 5 | 5 |
| PE_2.2 (reps 2,3) | 10 | 9 | 3 | 1 | 10 |
| **Exp 4** | **DEGs** | **True positives** | **True positives (%)** | **False positives** | **False positives (% SE)** |
| PE (3 reps) | 7639 | 7639 |  | 0 |  |
| SE (3 reps) | 7572 | 7455 | 98 | 117 | 2 |
| PE_2 (reps 1,2) | 7134 | 6788 | 89 | 346 | 5 |
| PE_2.2 (reps 2,3) | 6354 | 6255 | 82 | 99 | 2 |

Venn diagrams are presented in Figure S1 comparing the DEGs found using PE sequencing with 3 replicates (our gold standard in this experiment) and SE sequencing with 3 replicates versus using only 2 replicates from the PE data set.

**Figure S1. Differentially expressed genes (DEGs) identified in each of the four experiments using 2 biological replicates with PE or 3 biological replicates with SE.** Venn diagrams of the DEGs comparing those derived from using 3 biological replicates from the SE data (SE: purple) with 3 biological replicates from the PE data ( PE: dark orange) and using 2 biological replicates from the PE data (PE_2: light orange) Differentially expressed genes were identified using voom (limma) with an FDR cut-off of 0.05. (A-D) PE_2 uses replicate 1 and 2 of the PE data set (E-H) PE_2 uses replicate 2 and 3 of the PE data set.
