# Supplementary figures and images for: Differentially expressed genes from RNA-Seq and functional enrichment results are affected by the choice of single-end versus paired-end reads and stranded versus non-stranded protocols
Source: BMC Genomics. 2017 May 23;18:399. doi: 10.1186/s12864-017-3797-0 (PMC5442695; doi:10.1186/s12864-017-3797-0)

## Slide 1
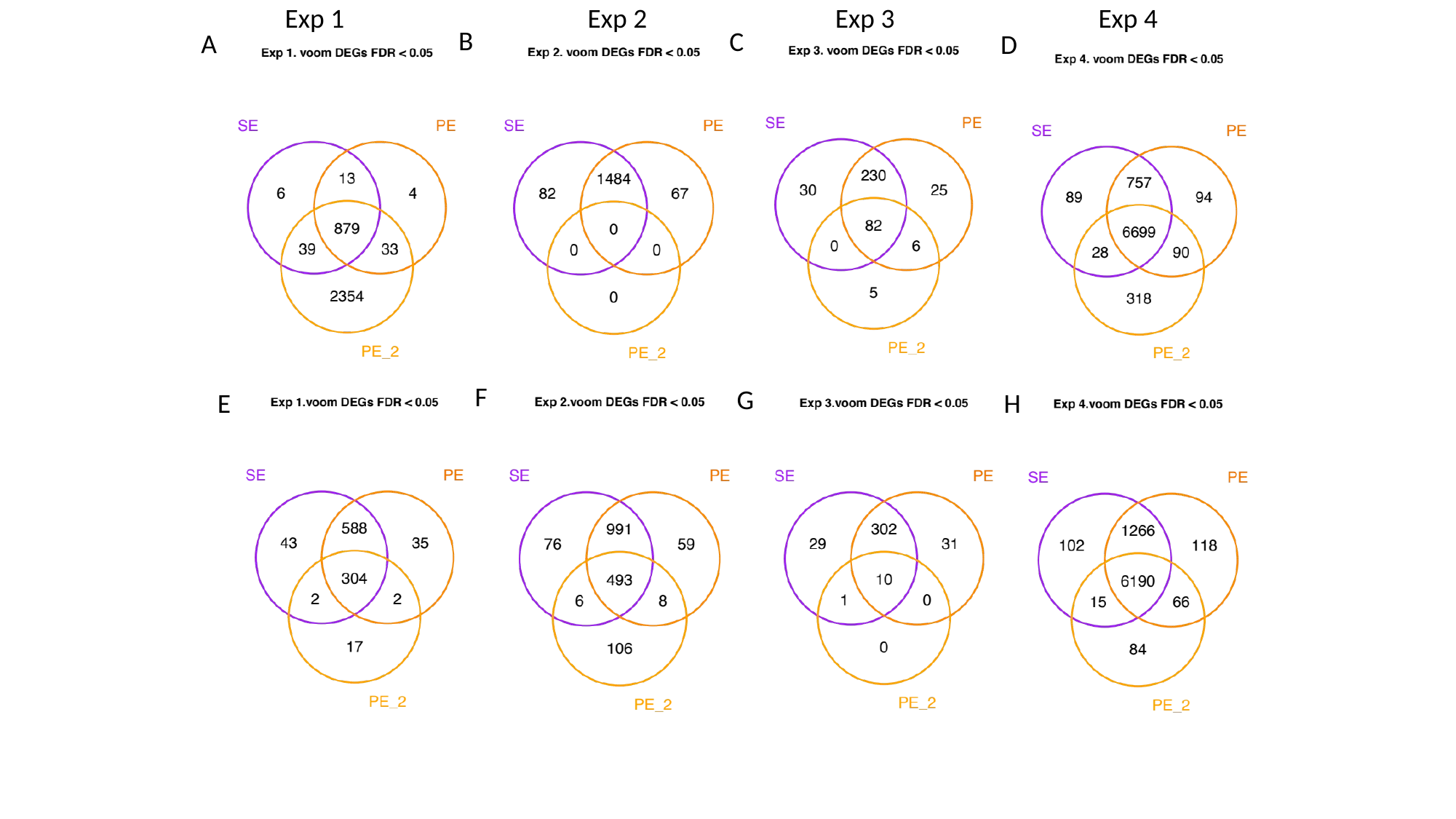

Exp 1
Exp 2
Exp 3
Exp 4
B
C
A
D
F
G
E
H

Supplement: Supplementary file 3 — Venn diagrams of the DEGs comparing 3 biological replicates from the SE data with 2 biological replicates from the PE data. (PPTX 237 kb) [file 12864_2017_3797_MOESM3_ESM.pptx]
